# Consumer perception of food variety in the UK: an exploratory mixed-methods analysis

**DOI:** 10.1186/s12889-020-09548-x

**Published:** 2020-09-24

**Authors:** Rochelle Embling, Aimee E. Pink, Michelle D. Lee, Menna Price, Laura L. Wilkinson

**Affiliations:** 1 Department of Psychology, College of Human and Health Sciences, Swansea University, Swansea, SA2 8PP UK; 2 School of Social Sciences, Nanyang Technological University, Singapore, 639818 Singapore

**Keywords:** Food, Diet, Variety, Consumer understanding, Qualitative, Dietary guidelines

## Abstract

**Background:**

‘Food variety’ is a key term that is frequently used in dietary guidelines around the world. Consuming a variety of foods – be it within a meal, across meals, or as part of the whole diet – is one factor that has been shown to increase food intake. However, little is known about consumer understanding of variety, and this may be a potential barrier to the success of dietary guidelines in today’s ‘obesogenic’ environment. This research sought to explore 1) consumer recognition of different forms of variety, and 2) consumer definitions of variety.

**Methods:**

In an online study (*N* = 240), participants were asked to discuss a range of photographs depicting different forms of variety, and to directly define the term ‘food variety’. They were unaware of the research aim.

**Results:**

Using a mixed methods approach, directed content analysis of these data showed that individuals referenced multiple forms of variety in the presence of food photographs. However, when asked to define variety, participants tended to only discuss variety in the context of the whole diet.

**Conclusions:**

These findings emphasise a need to educate consumers about variety to encourage adherence to dietary guidelines and help consumers better manage their own food intake.

## Background

‘Food variety’ is a key term that is frequently present in dietary guidelines around the world [1, 2]. Food variety is essential for ensuring adequate human nutrition [3] and is a key driver of food intake [4]. Moreover, greater dietary variety of ‘energy-dense or non-recommended’ foods is also associated with increased body adiposity and overweight [5, 6]. Despite these influences of food variety on our health, little is known about consumers’ understanding of this concept [7].

Food variety is a concept that is thought to be poorly defined, even within the research literature [7]. A recent review has identified the predominant features that should be considered when defining variety [7]. First, consistent with a previous taxonomy of variety [8], it is suggested that variety should be defined by period; different foods can be consumed as part of the overall diet, different foods can be included in meals consumed within or across days, and different foods can be eaten within a single eating session. Second, the characteristics that constitute variety should be identified; variety may be defined as consuming foods from between or within food groups, as consuming foods that vary in energy or nutrient density, or as consuming foods that differ in their sensory characteristics. For this reason, Raynor and Vadiveloo also identify that variety can refer to a single food item if it consists of a combination of different sensory characteristics [7]. The prevailing view is that variety in the forms described above disrupt the process of sensory specific satiety, the gradual decline in food palatability that occurs for an eaten food relative to uneaten foods, as a consequence of habituation [9–12].

Given the lack of clarity around the term food variety in the research literature, we suggest that the clarity of the consumer understanding of this term is considered, especially when it is prominent in global dietary guidelines [1, 2]. Indeed, research suggests that consumers are uncertain of specialised terms present in advice relating to food choice, weight, and serving size recommendations, including ‘variety or balance’ [13]. For example, in one study, individuals often mentioned phrases present in dietary guidelines when asked to discuss health campaigns (e.g. ‘5-a-day’), but they were uncertain about how to follow advice in their own diet [14]. Moreover, prior research suggests that the consumer perception of variety differs to conceptualisations of variety in the literature. For example, studies have reported poor associations between the presence of components used to quantify variety within a meal – such as food groups, colours, and shapes - and participants’ subjective ratings of variety [15, 16]. This is despite evidence that individuals seem to have few difficulties identifying individual components of variety. For instance, participants were able to correctly categorise mixed dishes into food groups in one study [17], and participants referred to variety as a reason for consuming more chocolate when it consisted of multiple colours [18]. Individuals have even been shown to ‘anticipate’ the variety effect when selecting the courses of a hypothetical meal designed to constitute variety [19].

It is unclear why this mismatch between findings exists, therefore in this mixed methods study we aimed to gain a deeper nuanced understanding of consumer perception of food variety. We gathered text responses via an online questionnaire to determine 1) whether consumers were able to recognise different forms of variety, and 2) how consumers explicitly defined food variety.

## Method

### Participants

Participants (*N =* 240) were recruited online via Swansea University’s participant subject pool, social media, survey sharing platforms (e.g. ‘Survey Circle’), and the online participant database ‘Prolific’ [20]. Following guidelines by Tran, Porcher, Falissard, and Ravaud [21], it was estimated that 150 responses to the online survey were required to reach data saturation, and data collection was stopped after 357 responses had been recorded, to account for unusable data (e.g. where participants did not complete open-ended questions about variety and where duplicate responses were identified for the same participant). Participants were included if they lived in the UK, had self-assessed normal/corrected-to-normal vision, and were 18 years old or older. They were excluded if they had studied eating behaviour as part of a final year Undergraduate or Masters course module. They were also excluded if they had a current or historical diagnosis of eating disorders. See Supplemental Fig. 1 for a participant flowchart. Participants were compensated for their time with a payment of £2.50 via Prolific (in line with their guidelines on ethical payment of participants) or course credit on the local subject pool. No other compensation was given. Written informed consent was obtained from all participants. The study was approved by the Department of Psychology Research Ethics Committee, and data collection and analysis methods were preregistered with the Open Science Framework (OSF [22];). This research is reported in line with the Standards for Reporting Qualitative Research (SRQR) guidelines [23]; see the supplementary materials (‘Additional file 1’) for a checklist.

### Stimuli

Participants were presented with photographs to encourage the ‘spontaneous’ recognition of each form of variety that was identified in the literature (see Table 1 for details of photographs used). Where relevant, information that would allow participants to identify a specific outlet used was removed from images (e.g. supermarket logos, store signs). All foods and products shown in photographs were chosen on the basis that they would be familiar to UK consumers. Where relevant, foods were photographed on a white dinner plate against a white background from a top-down view using a high-resolution digital camera, and similar portions of foods were presented in each series of images. All photographs were edited using Microsoft Photos for Windows 10 and PhotoScape v3.7. All photographs were approximately 210 × 297 mm and displayed in colour.

**Table 1:** Summary of topics to be explored using each series of photographs

Series^a^	Photographs presented	Topic ^b^	Survey questions
1	Supermarket Aisles displaying snack foods	Recognition of the availability of different brands and varieties for a food item.	How would you describe the setting in each of these images?
			What comparisons can you make between these images?
2	Main meals (chicken chow mein, paella)	Recognition of across-meal variety (when having meals in a single day), and recognition of within-meal variety (when having a first and second course)	Please select the two meals that you would prefer.
	Desserts (lemon tart, vanilla cheesecake)		
			Please explain why you have chosen these two meals.
3	Main meals with a varied proportion of the same two foods (fries and salad)	Recognition of within-meal variety (when having a combination of different foods within a single course)	What comparisons can you make between the images?
4	A ‘low variety’ savoury food (margherita pizza)	Recognition of within-meal variety (when having a combination of different sensory components within a single food item)	Which of the meals/foods would you prefer? Why?
	A ‘high variety’ savoury food (Mediterranean vegetable pizza)		
			Which of the meals/foods would you expect to be more filling? Why?
5	A ‘low variety’ sweet food (vanilla cheesecake)	Recognition of within-meal variety (when having a combination of different sensory components within a single food item)	
	A ‘high variety’ sweet food (chocolate, toffee & honeycomb cheesecake)		
			Which of the meals/foods displayed above would you expect to like more? Why?
6	Single product consisting of assorted chocolates presented with two labels ^c^; a ‘low variety’ description on packaging (‘*Chocolates*’), and a ‘high variety’ description on packaging (‘*Caramel chocolate buttons and milk chocolates with soft toffee, soft caramel, crisp biscuit and cereal centers*’)	Recognition of variety within a single food item from an ingredient-focussed product description	Which label makes the food sound most appealing to you? Why?
			Which label makes the food sound more filling to you? Why?
			Which label is most likely to draw your attention to the product? Why?

^a^ Photographs were presented to participants in distinct series, i.e. participants would discuss the images in series 1 before moving on to discuss the images in series 2

^b^ Though photographs were chosen with a specific subcategory of variety in mind, it was expected that participants would naturally include other categories of variety that were related to the image shown in responses

^c^ The same product was shown in both photographs

### Procedure

Participants were directed to complete the study on Qualtrics. Participants were informed that the aim of the research was to “understand the factors that influence food choices in the supermarket”. They were presented with an information sheet and completed a consent form. Participants were then presented with each series of photographs in turn. They were asked to describe and compare the photographs in each series, and to justify their food preferences and expectations about their liking and the expected fullness of foods. After all food photographs had been displayed, participants were asked to directly define ‘food variety’. After completing the main portion of the study, participants provided demographic information including their age, gender, ethnicity, and occupation. Following Gatzemeier, Price, Wilkinson, and Lee [24], to characterise the sample, participants also completed the three-factor eating questionnaire-R18 (TFEQ-R18) as a measure of general eating habits [25]. Height and weight were self-reported. At the end of the study, participants were asked to “please tell us what you think the aim of this experiment to be” and provided with an opportunity to answer in an open-text field to evaluate potential demand awareness, before they were presented with a debrief form. The study was completed in approximately 30 min.

### Data analyses

#### Qualitative coding and analysis

In order to capture whether participants’ recognition of variety aligned with existing ideas about the topic from the research literature, we used directed content analysis to code qualitative data [26]. This allowed us to consider the consumer perspective on variety through the lens of the research literature, whilst still retaining some flexibility in our approach to code relevant data into new categories outside of a pre-existing framework (in this case, the categories of variety identified in past research). A formative categorization matrix was created and agreed upon by the first and last author before data analysis began. Generic categories were deduced based on the different forms of food variety that were identified in the extant literature. A theoretical definition of each form of variety was developed and a set of coding rules was specified to guide the analysis and improve objectivity when coding. Following Raynor and Vadiveloo [7], we focused here on distinguishing categories of variety by period. As such, we identified six distinct subcategories of variety. In the context of the whole diet, we distinguished the availability of different varieties and brands for a single food item [27] and the consumption of different foods across or within food groups [5] as subcategories of variety. In the context of having variety across meals, we distinguished the consumption of different foods as part of a single eating session (i.e. breakfast, lunch, dinner, or snacks) or multiple eating sessions as a subcategory of variety [28]. In the context of a single eating session, we distinguished the presence of different foods across the successive courses of a meal [4, 29], different food components as part of a single course [30], and a combination of different sensory characteristics within a single food item [18, 29] as subcategories of variety. See the supplementary materials (‘Additional file 1’) for full definitions and coding rules for each category included in the formative categorization matrix.

In line with this approach, responses to each question were thoroughly read, and all statements from participants that were relevant to the subject matter were selected and given preliminary codes. Codes were then grouped according to their meanings, similarities and differences, and sub-categorised. Broader themes were identified using an inductive approach. All data was anonymised from the outset.

#### Quantitative analysis of frequency of codes

The number of participants that recognised each form of variety within broader themes was recorded to allow for a quantitative description of the occurrence of themes. To enhance trustworthiness, an independent researcher coded a random sample of statements using the formative categorisation matrix (they assigned predefined categories of variety to approximately 10% of all statements recorded). Following McAlister et al. [31], inter-rater reliability was calculated as the number of agreed codes divided by the total number of codes assigned to statements and converted to a percentage. Disagreements based on interpretations of predefined categories were discussed at intervals and coders reassessed statements independently. Agreement was found in 81% of cases. Where changes to the codebook had been decided as a result of discussions, coding for all data was adjusted where necessary, for both qualitative and quantitative analyses.

### Pilot study

Before collecting data for the main study, a pilot study (*N* = 22) was conducted to test the feasibility of this qualitative approach (see materials on the OSF for a description of data collection and analysis methods, and results of the pilot study [32];).

A series of six semi-structured focus groups, consisting of 2–5 participants, were conducted at Swansea University between November 2018 and February 2019. Following the same procedure as the main study, participants were unaware that the topic of discussion was ‘food variety’, food photographs were used as prompts to encourage the ‘spontaneous’ recognition of each form of variety that was identified in the literature, and participants were asked to directly define ‘food variety’. Using directed content analysis and the framework described above, results showed that participants’ discussion of the topic was consistent with categories recognised in the research literature when presented with food photographs. These preliminary results support the use of the formative categorisation matrix that we developed, as well as the stimuli and questions that we selected; statements referring to variety were captured by predefined categories, and no additional categories were identified. As such, we moved forward with this approach in the main study, and recruited a significantly larger sample.

## Results

### Participant characteristics

Participants included 138 females and 102 males; for 1 participant, their identified gender was not assigned at birth. Gender was unknown for 2 participants. See the supplementary materials (‘Additional file 1’) for details of location, ethnicity, and occupation. Mean scores on subscales of the TFEQ-R18 were comparable to the literature, falling just below the midpoint of each subscale [24, 33]. Most participants seemed to be unaware of the study aims; when reporting their beliefs about the aim of the study, five participants made a general reference to food variety, and one participant referred to an interest in defining variety. See Table 2.

**Table 2:** Sample characteristics (*N =* 240)

Demographics	Range	M (SD)
Age (years)	18–82	28.5 (12.2)
BMI (kg/m^2^)	16.3–40.7	24.4 (4.8)
Restraint ^a^	0.0–88.9	39.8 (20.4)
Uncontrolled eating ^a^	3.7–100.0	45.9 (18.8)
Emotional eating ^a^	0.0–100.0	44.4 (26.9)

^a^ Subscale score of the TFEQ; calculated by summing coded items for the respective subscale and transforming raw scores to a 0–100 scale (((raw score − lowest possible raw score)/possible raw score range) × 100)

### Main results

Directed content analysis showed that participants referred to six predefined categories of variety identified in the literature when presented with food photographs; dietary variety, brand variety, across-meal variety, variety between courses, variety within a single course, and variety within a single food. We also identified four broader themes relating to the context in which variety was recognised using an inductive approach; participants spontaneously referenced variety in food photographs, participants justified their food choices with reference to variety, participants justified their food expectations with reference to variety, and participants defined variety in accordance with our predefined categories. Consistent with the SRQR guidelines [23], these themes are discussed with reference to quotes from participants (participant IDs are given in brackets), and frequencies of themes. For an example of the data coding process, see Table 3.

**Table 3:** Example of coding for themes using a categorization matrix in the main study

Meaning unit	Summarized meaning unit	Codes	Predefined category of variety	Theme
“162 and 215 contains chocolate, although 162 contains a wide variety, while 215 contains mainly one brand.” (P9)	Variety of brands available	Differences in brand/ product availability	Brand variety	Spontaneously referring to variety
“I’d prefer to eat savoury food first and then sweet food. The savoury meal I chose looked the most appealing and the sweet food I [chose] looked the best.” (P37)	Preference for having savoury then sweet across meals	Preference for variety across meals	Across-meal variety	Justifying food choices with reference to variety
“435 it has a lot more going on lots of colors and textures which seems like it would be more filling” (P100)	Food more filling with more textures/colours	Variety in a food influences expected fullness	Variety within a food	Justifying food expectations with reference to variety
“I would define ‘food variety’ as a mixture of food groups; so having a balanced amount of each individual food [group] (e.g. carbohydrates, fats, dairy and vegetables etc.) but also different types of food within each food group; for example, take carbohydrates, not just eating bread but also pasta, rice and potatoes etc.” (P47)	‘Food variety’ is having a balanced diet of foods belonging to different food groups	Defining variety across the diet	Dietary variety	Defining variety

#### Theme 1 – spontaneously referring to variety

90% of participants referred to the presence of different ingredients and sensory characteristics within foods - such as different toppings, layers, flavours, colours, and textures - and emphasising these features on a product label seemingly increased the appeal of a product. References also often related to the presence of brand variety in supermarket aisles, as 68% of participants drew attention to branding and marketing features that differed between products. For instance, participants highlighted the different products and varieties of a product (relating to flavour) available within brands, and commented on differences in the organisation and packaging of products relating to price, colour, shape and portion size between brands.

P72: Image 435 is more colourful and appetising to look at. There's variety and flavour on that pizza, in comparison to image 127 that just appears bland and unappetising.

P58: First of all two pictures are sweet snacks, chocolates. They have all the good brands together and stock different flavours of the same brands just as the savoury, crisps shelves. However, on photo 081 compared to 295, there is not much colour for the crisps and more colour can be seen in the chocolate shelves.

In contrast, relatively fewer references were made to other categories of variety. Relating to dietary variety, 16% of individuals referred to broader differences between food items (e.g. ‘savoury versus sweet’ or ‘crunchy versus chewy’), the nutritional value of foods (e.g. high in sugar, salt and fat), or generally categorised items into a single group (e.g. ‘junk food’). Relating to variety within a single eating session, 8% of participants commented on the number of different colours, textures, foods, nutrients and ingredients included within a single course, whilst just 0.5% of participants mentioned variety across multiple courses.

P115: All items come out of the ground, vary in colour and texture, depending on how the chips are cooked picture 621 may not be as healthy to eat as 991.

P183: 819, 420 and 337 are healthier but they also only give an impression of healthiness because they are not nutritionally balanced. […] Something else would need to be added to make the meal satisfying (ie you won't feel hungry again very shortly afterwards) and also more nutritionally sound. It is also not very appealing to the eye as it is all the one colour, and therefore does not particularly whet the appetite.

#### Theme 2 – justifying food choices with reference to variety

When asked to justify their preferences for meals and foods displayed in images, the majority of participants referred to variety within meals. When choosing multiple courses, 54% of participants justified their choice with reference to variety; they often wanted and expected to have ‘a savoury followed by sweet course’, a main and dessert course, different flavours across courses, or simply different foods between courses. 74% of participants also preferred variety within a single course. In relation to variety within foods, 78% referred to variety when justifying their preference for one food or another; whilst some participants preferred a combination of different sensory characteristics and components within a food, others preferred to eat ‘plain’ or ‘simple’ foods that consisted of fewer flavours, textures and ingredients.

P128: After eating savoury food, it is nice to change to something sweet i.e. a dessert.

P165: 819 - because it contains 2 different things, so that [there] is different textures and tastes, I would find this more appealing [than] a plate that just had either chips, or just salad.

P169: I would prefer the food in the image 435 because I like it when a food has a variety of different ingredients.

In terms of having variety across multiple eating sessions, 36% of participants referred to variety across meals, whilst 2% referred to dietary variety. For example, participants wanted different sensory characteristics and preferred different foods across meals, though some participants referred to wanting ‘similar’ foods (e.g. two main courses/savoury dishes, or two sweet dishes). Some participants also justified their meal choices in relation to their current dietary needs and preferences.

P100: 338 and 202 look boring, not many colours that make it look appetising, also I wouldn't want to eat the same meal twice in one day.

P42: 621 because I like carbs but vegetables are something which I usually have to try and add in to my diet.

#### Theme 3 – justifying food expectations with reference to variety

When justifying food expectations, 88% of participants mentioned variety within foods. On the one hand, individuals believed that having multiple components and sensory characteristics within foods would increase their feeling of fullness and satisfaction after eating, and that emphasising variety on a food label made the food sound more filling because it highlights different ingredients, flavours and textures. Reasons included the beliefs that foods would have added nutrients, foods would have greater variety, and it would feel like they were eating more food. On the other hand, participants also suggested that they would feel fuller after eating foods with fewer components, and a general food label made the food sound more filling because it highlighted that the food was ‘plain’ and had less variety. Similar reasons were given by 23% of participants when justifying expectations for a single course.

P10: 435, because it has more flavours that way seemingly making it feel more filling, and also it most likely still has the same amount of cheese as 127, but additionally also has toppings.

P73: 435 does seem to contain more nutrients and taste. It may leave you wanting more, but overall presumably more filling.

P31: Ironically, despite having less ingredients on the pizza, i'd expect 127 to be more filling because it is just dough, cheese and tomato instead of a balance and mixture of many.

#### Theme 4 – directly defining ‘food variety’

When asked to directly define ‘food variety’, participants mentioned all six categories of variety that were predefined; dietary variety, brand variety, variety across meals, variety between courses, variety within a single course, and variety within a single food. However, it was notable that 88% of participants defined food variety with reference to dietary variety, including 6% who referred to brand variety. Definitions provided by participants typically related to the consumption of a range of foods belonging to different food groups as part of a balanced diet. In contrast, relatively fewer participants referred to other forms of variety; 5% mentioned across-meal variety, 1% variety across multiple courses, 2% variety within a single course, 1% variety within a single food item, and 4% referred to within-meal variety with no specification regarding a sub-category. Examples of definitions relating to each form of variety are included below:

P79: A good balance of food, from fruits and vegetables of a variety of colours, to grains and carbs, dairy, meat and protein and some sugary/high fat foods, similar to the food plate the government health guidelines used to promote.

P148: Having a wide variety of options ranging from different brands.

P131: Having 4 or more different meals a week.

P157: Food variety is, when I can choose from different mains, [different] appetisers and different [desserts], not only between [desserts] and mains for example.

P58: I would define food variety as having lots of different food groups in a meal and lots of different flavours, smells and textures.

P165: Food variety to me, means containing different ingredients, so a veggie pizza to me has a lot more variety than a cheese pizza because it has more than one item on it.

## Discussion

In this study, participants consistently discussed six categories of variety that were identified in the literature; dietary variety, brand variety, across-meal variety, variety between courses, variety within a single course, and variety within a single food. They also recognised each form of variety within the four main contexts; when spontaneously referring to variety in food photographs, when justifying food choices, when justifying food expectations, and when directly defining variety. The proportion of individuals recognising different forms of variety differed across these themes; despite referring to different forms of variety in the presence of food photographs, the majority of participants provided definitions of variety that related only to dietary variety.

These results further elucidate the findings of Hale and Varakin [18], who found that snack preference was not only influenced by the presence of colour variety within a food, but that participants justified their preference with reference to variety. Our research demonstrates that individuals may also consider variety when choosing meals and foods that consist of multiple components, and when they vary in more than one sensory characteristic. This supports variety as a factor that consumers actively consider when choosing foods. Previous research has shown that consumed portions are planned from the outset in 92% of cases [34], and variety is one factor that can increase selected portion size *before* eating [19]. Our research then suggests that consumers may be aware of the influence of variety on consumption when meal planning.

Our results also indicate that there was a significant difference in participants’ recognition of variety in the presence of food photographs compared to when asked to directly define the concept. This builds on the findings of prior research demonstrating that consumer ratings of variety within meals do not reflect the presence of components that are used to operationalise variety in studies [15, 16]. One explanation for this is that the appreciation of variety is a stimulus-driven response that requires little cognitive effort on behalf of the consumer. Wilkinson et al. [19] previously demonstrated that participants expected a course to be more pleasant and selected a larger portion if it was sensorially different to the previous course, and these decisions were made in approximately 15 s. This suggests that the appreciation of variety is a habitual response, and that asking participants to ‘abstractly’ define variety outside of the context of making food choices may be a difficult task.

An alternative explanation is that dietary guidelines oversimplify the presence of variety in the eating environment, and this was reflected in participants’ definitions of the term. Variety within food groups is often highlighted in the most recent dietary recommendations [2], and this is consistent with the finding that consumers are able to accurately categorise meals and foods into food groups [17]. However, little attention is given to the presence of variety within- and across-meals, the food components that constitute variety (i.e., sensory characteristics), nor the potential role of variety in encouraging greater food intake [4]. A recent scientific advisory article from the American Heart Association has identified the poor level of correspondence between the research literature and dietary advice as an issue that may undermine public health efforts to promote healthy eating patterns [35]. Our research further emphasises the need for dietary guidelines to discern the nature of variety to consumers in line with the research literature.

This research also affords an opportunity to improve dietary strategies focused on variety. Raynor and Vadiveloo [7] have recognised that the success of current interventions relies heavily on the ability of the consumer to identify, monitor and manage their own intake in the presence of multiple forms of variety. Previous studies have shown that cognitive strategies that use cues to manipulate the perception of variety within a meal to encourage the consumption of healthy foods and reduce the consumption of unhealthy foods are unsuccessful [36–38]. Our research suggests that this may be due to the consumer understanding of variety as a concept rather than a lack of awareness of variety, and that educating consumers about variety may improve the success of future priming strategies. For example, Epstein et al. [39] found that encouraging children and parents to limit variety (e.g. by planning to repeat meals, use leftovers, and choose only one snack food for the duration of the intervention), in conjunction with attending regular counselling meetings to support dietary changes, led to greater weight loss in a preliminary family intervention trial. However, the feasibility of such an approach in a real-world setting, for a longer period, warrants further exploration.

A strength of the current research is that, by using a mixed methods approach, we have explored consumer understanding of the topic of food variety in depth for the first time. Potential limitations that should be acknowledged firstly includes that using a directed approach to data analysis can increase the risk of bias when coding data, and the likelihood that results will be supportive of a given construct [40]. It was necessary to predefine categories of variety before data collection in order to select appropriate photographs to use in this research. However, measures were taken to improve the trustworthiness of our analyses. Results from the main study replicated results found in a pilot study, and an independent researcher coded a subset of the responses from Study 2 to confirm themes.

Second, the use of food photographs may be considered a prime or demand characteristic that would make it obvious to participants that the subject of interest was actually food variety, particularly as participants were asked to compare images that displayed either ‘high’ or ‘low’ variety meals and foods. In particular, the majority of questions and photographs presented to participants were most relevant to the discussion of variety within meals, and this may have increased the frequency of discussion of within-meal variety in the presence of food photographs. We reason that priming effects appear to be unlikely here, as participants often discussed factors unrelated to variety when justifying food preferences (e.g. general food liking, their familiarity with foods). Participant feedback also supports that the majority of participants were unaware of the study aims, and it remains notable that significantly fewer participants defined variety with reference to within-meal variety.

Third, the use of food photographs also means that the perception of variety was considered in response to hypothetical food choices rather than actual consumption. This is important to consider as the awareness of sensory characteristics unrelated to visual appearance – such as texture and smell - may have been reduced by using this approach. Though, we highlight that our results were consistent with Hale and Varakin [18], who measured participants’ awareness of variety after they had consumed foods in the laboratory. Nevertheless, it would be useful for future research to explore the perception of different forms of variety, and the experience of multiple sensory characteristics, in response to eaten foods that better reflect consumption in the real world.

## Conclusions

Taken together, this research demonstrates that consumers actively consider variety within meals, across meals, and in the context of the whole diet. Our results also highlight education as a potential tool to improve the consumer understanding of the concept of variety, bridging the gap between the conceptualisation of variety in the literature and the presence of different forms of variety in the eating environment to help consumers follow dietary advice and manage their own food consumption.

## Supplementary information

**Additional file 1.** Supplementary materials include the completed SRQR reporting checklist for qualitative research, the formative categorization matrix used in this research, supplementary participant demographics, and a participant flow chart.

## Data Availability

The datasets generated and analysed are available from the corresponding author on reasonable request.

## Abbreviations

TFEQ-R18: Three-factor eating questionnaire-R18
SRQR: Standards for Reporting Qualitative Research guidelines

## References

[1] Kennedy E (2004). Dietary diversity, diet quality, and body weight regulation. Nutr Rev [Internet].

[2] Vadiveloo MK, Parekh N (2015). Dietary variety: an overlooked strategy for obesity and chronic disease control. Am J Prev Med.

[3] Nair MK, Augustine LF, Konapur A (2016). Food-based interventions to modify diet quality and diversity to address multiple micronutrient deficiency. Front Public Health.

[4] Rolls BJ, Rowe EA, Rolls ET, Kingston B, Megson A, Gunary R (1981). Variety in a meal enhances food intake in man. Physiol Behav.

[5] McCrory MA, Fuss PJ, McCallum JE, Yao MJ, Vinken AG, Hays NP (1999). Dietary variety within food groups: association with energy intake and body fatness in men and women. Am J Clin Nutr [Internet].

[6] Vadiveloo M, Dixon LB, Parekh N (2013). Associations between dietary variety and measures of body adiposity: a systematic review of epidemiological studies. Br J Nutr [Internet].

[7] Raynor HA, Vadiveloo M (2018). Understanding the Relationship Between Food Variety, Food Intake, and Energy Balance. Curr Obes Rep [Internet].

[8] Meiselman HL, deGraaf C, Lesher LL (2000). The effects of variety and monotony on food acceptance and intake at a midday meal. Physiol Behav [Internet].

[9] Brondel L, Romer M, Van Wymelbeke V, Pineau N, Jiang T, Hanus C (2009). Variety enhances food intake in humans: Role of sensory-specific satiety. Physiol Behav [Internet].

[10] Epstein LH, Fletcher KD, O’Neill J, Roemmich JN, Raynor H, Bouton ME (2013). Food characteristics, long-term habituation and energy intake. Laboratory and field studies. Appetite [Internet].

[11] Raynor HA, Niemeier HM, Wing RR (2006). Effect of limiting snack food variety on long-term sensory-specific satiety and monotony during obesity treatment. Eat Behav.

[12] Rolls BJ, Rolls ET, Rowe EA, Sweeney K (1981). Sensory specific satiety in man. Physiol Behav.

[13] Brown KA, Timotijevic L, Barnett J, Shepherd R, Lahteenmaki L, Raats MM (2011). A review of consumer awareness, understanding and use of food-based dietary guidelines. Br J Nutr.

[14] Khanom A, Hill RA, Morgan K, Rapport FL, Lyons RA, Brophy S (2015). Parental recommendations for population level interventions to support infant and family dietary choices: a qualitative study from the growing up in Wales, environments for healthy living (EHL) study.

[15] Haugaard P, Brockhoff PB, Lähteenmäki L, Lahteenmaki L, Lähteenmäki L, Lahteenmaki L (2016). Objective measures of meal variety lacking association with consumers’ perception of variety with self-selected buffet meals at work. Food Qual Prefer [Internet].

[16] König LM, Renner B, Koenig LM, Renner B, König LM, Renner B (2018). Colourful = healthy? Exploring meal colour variety and its relation to food consumption. Food Qual Prefer [Internet].

[17] Britten P, Haven J, Davis C (2006). Consumer Research for Development of Educational Messages for the MyPyramid Food Guidance System. J Nutr Educ Behav [Internet].

[18] Hale J, Varakin AD (2016). Awareness of the influence a variety of food has on food consumption. N Am J Psychol.

[19] Wilkinson LL, Hinton EC, Fay SH, Rogers PJ, Brunstrom JM (2013). The “variety effect” is anticipated in meal planning. Appetite [Internet].

[20] Prolific [Internet]. [cited 2020 Sep 7]. Available from: https://www.prolific.co/.

[21] Tran V-T, Porcher R, Falissard B, Ravaud P (2016). Point of data saturation was assessed using resampling methods in a survey with open-ended questions. J Clin Epidemiol.

[22] Embling R, Pink AE, Lee M, Price M, Wilkinson LL. Do consumers in the UK recognise “food variety” in the everyday eating environment? Online Qual Study [Internet]. 2020; Available from: https://osf.io/5etd4/, [cited 2020 Sep 7].

[23] O’Brien BC, Harris IB, Beckman TJ, Reed DA, Cook DA (2014). Standards for Reporting Qualitative Research. Acad Med [Internet].

[24] Gatzemeier J, Price ML, Wilkinson L, Lee M (2019). Understanding everyday strategies used to manage indulgent food consumption: A mixed-methods design. Appetite [Internet].

[25] Karlsson J, Persson L-O, Sjöström L, Sullivan M (2000). Psychometric properties and factor structure of the three-factor eating questionnaire (TFEQ) in obese men and women. Results from the Swedish obese subjects (SOS) study. Int J Obes.

[26] Assarroudi A, Nabavi FH, Armat MR, Ebadi A, Vaismoradi M (2018). Directed qualitative content analysis: the description and elaboration of its underpinning methods and data analysis process. J Res Nurs.

[27] Hardman CA, Ferriday D, Kyle L, Rogers PJ, Brunstrom JM. So Many Brands and Varieties to Choose from: Does This Compromise the Control of Food Intake in Humans? Brown A, editor. PLoS One [Internet]. 2015;10(4):e0125869. Available from: %3CGo. 10.1371/journal.pone.0125869 PMC4414581 25923118

[28] Haws KL, Liu PJ, Redden JP, Silver HJ (2017). Exploring the relationship between varieties of variety and weight loss: When more variety can help people lose weight. J Mark Res [Internet].

[29] Rolls BJ, Rowe EA, Rolls ET (1982). How sensory properties of foods affect human feeding behavior. Physiol Behav [Internet].

[30] Wijnhoven HA, van der Meij BS, Visser M (2015). Variety within a cooked meal increases meal energy intake in older women with a poor appetite. Appetite [Internet].

[31] AM MA, Lee DM, Ehlert KM, Kajfez RL, Faber CJ, Kennedy MS (2017). Qualitative Coding: An Approach to Assess Inter-Rater Reliability.

[32] Embling R, Pink AE, Lee M, Price M, Wilkinson LL (2020). Do consumers in the UK recognise “food variety” in the everyday eating environment? A focus group study [Internet].

[33] Price M, Higgs S, Lee M (2015). Self-reported eating traits: underlying components of food responsivity and dietary restriction are positively related to BMI. Appetite..

[34] Fay SH, Ferriday D, Hinton EC, Shakeshaft NG, Rogers PJ, Brunstrom JM (2011). What determines real-world meal size? Evidence for pre-meal planning. Appetite..

[35] de Oliveira Otto MC, Anderson CAM, Dearborn JL, Ferranti EP, Mozaffarian D, Rao G (2018). Dietary diversity: implications for obesity prevention in adult populations: a science advisory from the American Heart Association. Circulation..

[36] Embling R, Price M, Lee M, Wilkinson L (2019). Food-variety-focused labelling does not increase ideal portion size, expected fullness or snack intake. Food Qual Prefer [Internet].

[37] Friis R, Skov LR, Olsen A, Appleton KM, Saulais L, Dinnella C, et al. Comparison of three nudge interventions (priming, default option, and perceived variety) to promote vegetable consumption in a self-service buffet setting. Kunze G, editor. PLoS One [Internet]. 2017;12(5):e0176028. Available from: https://www.cochranelibrary.com/central/doi/10.1002/central/CN-01784726/full. 10.1371/journal.pone.0176028 PMC5450998 28562678

[38] Vadiveloo M, Principato L, Morwitz V, Mattei J (2019). Sensory variety in shape and color influences fruit and vegetable intake, liking, and purchase intentions in some subsets of adults: A randomized pilot experiment. Food Qual Prefer [Internet].

[39] Epstein LH, Kilanowski C, Paluch RA, Raynor H, TO D (2015). Reducing variety enhances effectiveness of family-based treatment for pediatric obesity. Eat Behav [Internet].

[40] Hovbrandt P, Håkansson C, Albin M, Carlsson G, Nilsson K (2019). Prerequisites and driving forces behind an extended working life among older workers. Scand J Occup Ther.

