# Supplementary material for: Consumer perception of food variety in the UK: an exploratory mixed-methods analysis
Source: BMC Public Health. 2020 Sep 24;20:1449. doi: 10.1186/s12889-020-09548-x (PMC7517603; doi:10.1186/s12889-020-09548-x)
Supplement: Supplementary file 1 — Additional file 1. Supplementary materials include the completed SRQR reporting checklist for qualitative research, the formative categorization matrix used in this research, supplementary participant demographics, and a participant flow chart. [file 12889_2020_9548_MOESM1_ESM.docx]

**Supplementary Material**

**Consumer perception of food variety in the UK: An exploratory mixed-methods analysis**

Rochelle Embling^1^, Aimee E. Pink^1,2^, Michelle Lee^1^, Menna Price^1^, & Laura L. Wilkinson^1^

^1^Department of Psychology, College of Human and Health Sciences, Swansea University, Swansea, SA2 8PP, UK.

^2^School of Social Sciences, Nanyang Technological University, Singapore, 639818.

**Description of supplemental materials:**

**Supplemental Table 1** SRQR reporting checklist for qualitative research

**Supplemental Table 2** Formative categorization matrix

**Supplemental Table 3** Supplementary participant demographics

**Supplemental Figure 1** Participant flow chart of study

**Online Supporting Material**

**Supplemental Table 1** SRQR reporting checklist for qualitative research^*^

|  |  | Reporting Item | Page Number |
| --- | --- | --- | --- |
| **Title** |  |  |  |
|  | [#1](about:blank#1) | Concise description of the nature and topic of the study identifying the study as qualitative or indicating the approach (e.g. ethnography, grounded theory) or data collection methods (e.g. interview, focus group) is recommended | 1 |
| **Abstract** |  |  |  |
|  | [#2](about:blank#2) | Summary of the key elements of the study using the abstract format of the intended publication; typically includes background, purpose, methods, results and conclusions | 2 |
| **Introduction** |  |  |  |
| Problem formulation | [#3](about:blank#3) | Description and significance of the problem / phenomenon studied: review of relevant theory and empirical work; problem statement | 3 – 4 |
| Purpose or research question | [#4](about:blank#4) | Purpose of the study and specific objectives or questions | 4 |
| **Methods** |  |  |  |
| Qualitative approach and research paradigm | [#5](about:blank#5) | Qualitative approach (e.g. ethnography, grounded theory, case study, phenomenolgy, narrative research) and guiding theory if appropriate; identifying the research paradigm (e.g. postpositivist, constructivist / interpretivist) is also recommended; rationale. The rationale should briefly discuss the justification for choosing that theory, approach, method or technique rather than other options available; the assumptions and limitations implicit in those choices and how those choices influence study conclusions and transferability. As appropriate the rationale for several items might be discussed together. | 6 – 9 |
| Researcher characteristics and reflexivity | [#6](about:blank#6) | Researchers' characteristics that may influence the research, including personal attributes, qualifications / experience, relationship with participants, assumptions and / or presuppositions; potential or actual interaction between researchers' characteristics and the research questions, approach, methods, results and / or transferability | 17-18 *(discussed as a potential limitation of the research)* |
| Context | [#7](about:blank#7) | Setting / site and salient contextual factors; rationale | 4 - 5 |
| Sampling strategy | [#8](about:blank#8) | How and why research participants, documents, or events were selected; criteria for deciding when no further sampling was necessary (e.g. sampling saturation); rationale | 4 - 5, and supplementary Figure 1 |
| Ethical issues pertaining to human subjects | [#9](about:blank#9) | Documentation of approval by an appropriate ethics review board and participant consent, or explanation for lack thereof; other confidentiality and data security issues | 4 - 5 |
| Data collection methods | [#10](about:blank#10) | Types of data collected; details of data collection procedures including (as appropriate) start and stop dates of data collection and analysis, iterative process, triangulation of sources / methods, and modification of procedures in response to evolving study findings; rationale | 5 – 9 |
| Data collection instruments and technologies | [#11](about:blank#11) | Description of instruments (e.g. interview guides, questionnaires) and devices (e.g. audio recorders) used for data collection; if / how the instruments(s) changed over the course of the study | 5 – 9, and supplementary materials |
| Units of study | [#12](about:blank#12) | Number and relevant characteristics of participants, documents, or events included in the study; level of participation (could be reported in results) | 4 - 5, 9 – 10, Supplementary Table 3 |
| Data processing | [#13](about:blank#13) | Methods for processing data prior to and during analysis, including transcription, data entry, data management and security, verification of data integrity, data coding, and anonymisation / deidentification of excerpts | 7 – 8 |
| Data analysis | [#14](about:blank#14) | Process by which inferences, themes, etc. were identified and developed, including the researchers involved in data analysis; usually references a specific paradigm or approach; rationale | 7 - 9, and supplementary materials |
| Techniques to enhance trustworthiness | [#15](about:blank#15) | Techniques to enhance trustworthiness and credibility of data analysis (e.g. member checking, audit trail, triangulation); rationale | 8, 17 - 18 *(considered in the discussion)* |
| **Results/findings** |  |  |  |
| Syntheses and interpretation | [#16](about:blank#16) | Main findings (e.g. interpretations, inferences, and themes); might include development of a theory or model, or integration with prior research or theory | 10 - 15 |
| Links to empirical data | [#17](about:blank#17) | Evidence (e.g. quotes, field notes, text excerpts, photographs) to substantiate analytic findings | 10 - 15 |
| **Discussion** |  |  |  |
| Intergration with prior work, implications, transferability and contribution(s) to the field | [#18](about:blank#18) | Short summary of main findings; explanation of how findings and conclusions connect to, support, elaborate on, or challenge conclusions of earlier scholarship; discussion of scope of application / generalizability; identification of unique contributions(s) to scholarship in a discipline or field | 15 - 19 |
| Limitations | [#19](about:blank#19) | Trustworthiness and limitations of findings | 17 - 18 |
| **Other** |  |  |  |
| Conflicts of interest | [#20](about:blank#20) | Potential sources of influence of perceived influence on study conduct and conclusions; how these were managed | 19 |
| Funding | [#21](about:blank#21) | Sources of funding and other support; role of funders in data collection, interpretation and reporting | 19 |

^*^Retrieved from: O'Brien BC, Harris IB, Beckman TJ, Reed DA, Cook DA. Standards for reporting qualitative research: a synthesis of recommendations. Acad Med. 2014;89(9):1245-1251.

**Online Supporting Material**

**Supplemental Table 2** Formative categorization matrix

| Main Concept Category | Anchor Sample (from meaning units)  ***Identified during analysis for pilot study*** | Subcategory | Definition | Coding Rules |
| --- | --- | --- | --- | --- |
| Dietary Variety | *“Um... yeah, similarly like you said the main food groups like the carbs um, yeah protein’s another one and... my diet’s not great... so I should pay more attention to what I eat but for me variety is about balancing... it’s about balance it’s not about eating all carbs all the time it’s about having a bit of fruit, bit of veg, bit of carbs, bit of protein so having that balance for me yeah”*  *FG5, lines 675 – 684 (when defining variety)* | - Directly identifying food groups - Discussing the consumption of a range of foods across the diet | Reference should be made to the presence of a variety of foods across the diet; this may be in the form of dietary food groups (e.g. Dairy versus meat), broader categories of foods (e.g. sweet versus savoury), or macronutrients (e.g. carbohydrates versus protein)  “Generally consuming foods from different food groups, and different foods within food groups, (over a longer period of time)” | - Participants may directly reference variety without referring specifically to differences being present (e.g. they may refer to only eating sweet snacks, or that an image displays only savoury foods) - Codes must be specific to the definition - some form of food group should be mentioned, i.e. identifying only the food item (chocolate/crisps) should not be coded as an identification of dietary variety |
| Brand Variety | *“I’m just looking to see if there’s sort of more variety in the brands comparing the chocolate with the crisps and... I suppose with the [popular brand name] chocolate you’ve got... like varieties within the same brand […] And... um, but I suppose the... sort of number of brands might be the same between the crisps and uh, and the chocolates perhaps*  *FG6, lines 149-156* | - Directly identifying brands - Indirectly referring to brands | Reference should be made to the availability of different brands for a food item; this may be in the form of identifying examples of brands or features of branding (e.g. referring to differences in logos, colours of packaging, packaging size)  “The availability of different brands for a food item, including varieties that can vary in energy density and sensory characteristics” | - Participants may directly reference variety without referring specifically to differences being present (e.g. they may refer to there being lots of products available for a specific brand, or to having a preference for one brand over another) - Participants may indirectly reference the presence of different brands by referring more generally to packaging (e.g. similarities/differences in package size, package colours) – if in this instance no direct reference is made to brands, codes should be sub-categorised as an indirect reference to brand variety |
| Variety across meals | *“Like I wouldn’t have pasta salad for lunch and then... noodles for tea…*  *‘Cause they would be in the same group I try to like, just have one”*  *FG3, lines 568-570 (when defining variety)* | - Justifying food choices with reference to variety | Reference should be made to the availability of a variety of foods across meals when justifying food choices, e.g. having different foods for breakfast, lunch and dinner. This may be in the form of sensory characteristics (flavour, colour etc.), or broader food groups (desserts versus mains, sweet versus savoury)  “Consuming foods that differ in their sensory characteristics across-meals, be it within a single day or across multiple days.” | - Participants may directly reference variety (or lack thereof) when justifying their meal preferences (e.g. that they prefer chow mein and cheesecake because it’s savoury followed by sweet) - Codes must be specific to variety across meals – if participants refer to sensory characteristics/broader food groups in isolation, it should not be coded as a reference to variety (e.g. I like cheesecake because it’s sweet) |
| Variety between courses | *“Okay. Um, yeah, I’d do the same again, 467 [Paella dish] and 338 [Vanilla Cheesecake] …‘cause I just like savoury followed by sweet. I’d always have savoury first… Don’t know why*  *FG1, lines 146-152* | - Justifying food choices with reference to variety | Reference should be made to the availability of a variety of foods across the courses of a meal when justifying food choices, e.g. having different foods for a first and second course in a single eating sesson. This may be in the form of sensory characteristics (flavour, colour etc.), or broader food groups (desserts versus mains, sweet versus savoury).  “Consuming foods that differ in their sensory characteristics across the courses of a meal” | - Participants may directly reference variety (or lack thereof) when justifying their course preferences (e.g. that they prefer chow mein and cheesecake because it’s savoury followed by sweet) - Codes must be specific to variety across courses – if participants refer to sensory characteristics/broader food groups in isolation, with no inference of this being different across foods, it should not be coded as a reference to variety (e.g. I like cheesecake because it’s sweet) |
| Variety within a single meal (single course) | *“I’d want more variety [than chips and salad]...*  *Um as I said more including more... types of foods so you’re getting both nutritional value and... you know, enjoying it a bit more with different tastes”*  *FG6, lines 438 - 441* | - Directly identifying different foods in a meal - Justifying food choices with reference to variety - Identifying variety as an influencing factor on the expected liking of a meal - Identifying variety as an influencing factor on the expected fullness of a meal | Reference should be made to the presence of different foods in a single meal; this may be in the form of simply identifying the presence of different components in a meal, or different sensory characteristics in a meal (flavours/colours etc.).  **Note:** A single course is defined here as a course that contains multiple individual food items. E.g., Salad and Fries are two separate components of a single course.  “Consuming foods that differ in their sensory characteristics as part of a single course” | - Participants may refer to variety (or lack thereof) when describing/choosing meals - Codes must be specific to variety in a meal – if participants refer to sensory characteristics/meal components in isolation, with no inference of this being variable in the meal, it should not be coded as a reference to variety (e.g. I like meal X because it’s savoury) |
| Variety within a food | *“You got to have, yeah some different colours in [the salad] and some different flavours so when it’s just, ooh sorry. When it’s just lettuce and rocket it’s the same texture, it’s the same flavours, it’s the same colours. Yeah, if you put some tomatoes and olives, some cucumber and celery and like red and yellow peppers, that would be good ‘cause you’ve got a different kind of textures and you’ve got the flavours, and you’ve got the appearance”*  *FG2, lines 330-334* | - Directly identifying different components in a food - Justifying food choices with reference to variety - Identifying variety as an influencing factor on the expected liking of a food - Identifying variety as an influencing factor on the expected fullness of a food | Reference should be made to the presence of different sensory components within a food; this may be done directly (in terms of having different flavours/textures etc.) or by identifying food elements that infer differences in sensory characteristics (in terms of the food having different toppings, fillings etc.).  **Note:** A single food is defined here as an item that is popularly viewed as a whole, individual food component. E.g., Chicken Chow Mein is a single food item with multiple ingredients (chicken, noodles, peppers etc.), rather than a single course composed of multiple foods (chicken with noodles and peppers etc.).  “Consuming a food that contains a combination of different sensory characteristics in and of itself” | - Participants may refer to variety (or lack thereof) when describing/choosing the foods - Codes must be specific to variety in a food – if participants refer to sensory characteristics/food components in isolation, with no inference of this being variable in the food, it should not be coded as a reference to variety (e.g. I like food X because it’s sweet) |
| Variety in a product description^*^  ***(not a main category – defined here for distinction when coding)*** | *N/A* | - Appeal of ‘variety labelling’ - Influence of ‘variety labelling’ on expected fullness of a product - Conspicuousness of ‘variety labelling’ | ‘Variety labelling’ refers here to a description of a product that draws attention to variety within a food by listing different sensory components (flavours, textures etc.). | - When justifying the appeal/effect/conspicuousness of labels, participants may refer to variety (or lack thereof) in terms of sensory or food components - Codes must be specific to variety in a food – if participants refer to sensory characteristics/food components in isolation, with no inference of this being variable in the food/description, it should not be coded as a reference to variety |
| Defining food variety^*^  ***(not a main category – defined here for distinction when coding)*** | *N/A* | - Dietary variety - Brand variety - Variety across meals - Variety across courses - Variety within a meal (having different foods within a single course) - Variety within a food | Reference should be made to one or more of the subcategories (see previous for definitions of each) | - Note that context should be considered in relation to participant responses here as they may appear to talk about one form of food variety when they are actually talking about another e.g. they may discuss macronutrients as a form of food variety across the diet (as an alternative name to dietary food groups) or in terms of having different foods within a meal (e.g. ‘I like to have a bit of protein, some carbs and a vegetable for lunch’) |

^*^If coding meaning units after discussing variety on a label/defining variety, must be recognised in coding that answers were given in this context under the broader variety category

**Online Supporting Material**

**Supplemental Table 3** Supplementary participant demographics (*N* = 240)

| Demographics ​ | N |
| --- | --- |
| **UK location ​** |  |
| *England* | 139 |
| *Wales* | 72 |
| *Scotland* | 12 |
| *Northern Ireland* | 3 |
| *Unknown* | 14 |
| **Ethnicity** |  |
| *Caucasian* | 214 |
| *Asian/Asian British* | 17 |
| *Black/African/Caribbean/Black British* | 3 |
| *Mixed Race* | 2 |
| *Other* | 2 |
| *Unknown* | 2 |
| **Occupation** |  |
| *Student* | 118 |
| *In employment* | 103 |
| *Retired* | 4 |
| *Not otherwise in employment* | 8 |
| *Unknown* | 7 |

**Online Supporting Material**

**
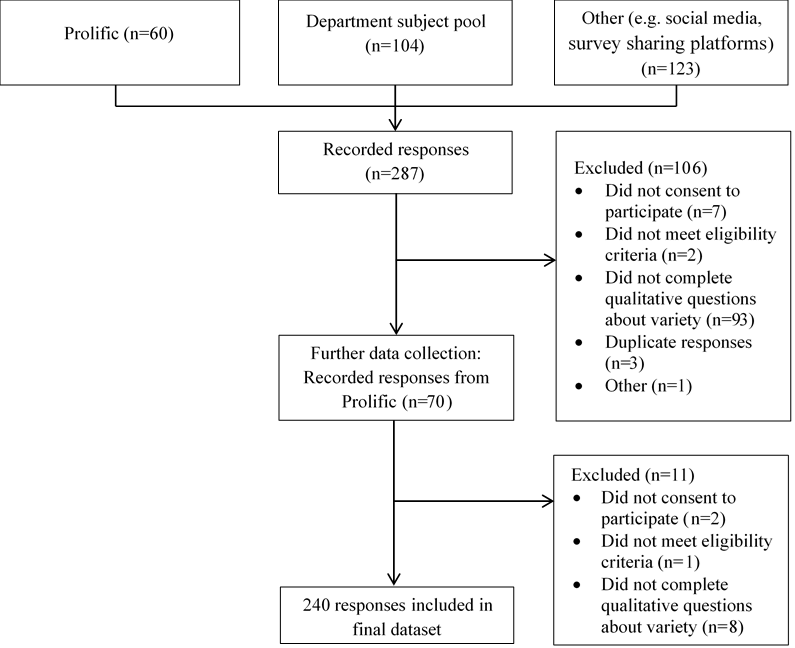
**

**Supplemental Figure 1**: Participant flow chart.
